# Surgical Treatment of Scaphoid Non-Union in Adolescents: A Modified Vascularized Bone Graft Technique

**DOI:** 10.3390/children12091135

**Published:** 2025-08-27

**Authors:** Diletta Bandinelli, Alessia Pagnotta, Alessandro Piperno, Martina Marsiolo, Angelo Gabriele Aulisa, Francesco Falciglia

**Affiliations:** 1 U.O.C. of Traumatology, Bambino Gesù Children’s Hospital, IRCCS, 00165 Rome, Italy; martina.marsiolo@opbg.net (M.M.); agabriele.aulisa@opbg.net (A.G.A.); francesco.falciglia@opbg.net (F.F.); 2 Hand and Microsurgery Unit, Jewish Hospital, 00148 Rome, Italy; a.pagnotta@ospedaleisraelitico.it (A.P.); a.piperno@ospedaleisraelitico.it (A.P.); 3 Department of Human Sciences, Society and Health, University of Cassino and Southern Lazio, 03043 Cassino, Italy

**Keywords:** scaphoid, non-union, graft, vascularized, bone graft, children, adolescents, fracture

## Abstract

**Background**: Although adolescents are at a lower risk of developing scaphoid non-union than adults, this complication is not uncommon in younger patients. The current gold standard for surgical treatment is non-vascularized bone grafting from the iliac crest or distal radius, and it is often considered the first-line option. However, non-union can persist in 10–20% of cases, and failure rates can reach up to 50% when the proximal pole is necrotic. **Methods**: We evaluated a modified vascularized bone graft surgical technique in selected adolescent patients, with the goal of avoiding growth-related complications. Our experience is based on three cases of scaphoid non-union treated surgically between June 2019 and June 2022. **Results and Conclusions**: The modified surgical technique has shown promising results in the selected cases and carries no risk of donor site morbidity. It enables preservation of severely compromised scaphoid bones, prevents early-onset wrist osteoarthritis, and facilitates the return to sports activities for young patients.

## 1. Introduction

The scaphoid is the most commonly fractured carpal bone. Scaphoid fractures in the pediatric population are relatively uncommon, representing 3% of hand fractures and 0.34% of all fractures [1].

There is little information in the literature regarding the epidemiology of scaphoid fractures in children, and even less data are available about non-union at this age. Historically, it was believed that most scaphoid fractures in children and adolescents involved the distal pole [2].

**Figure 1:**
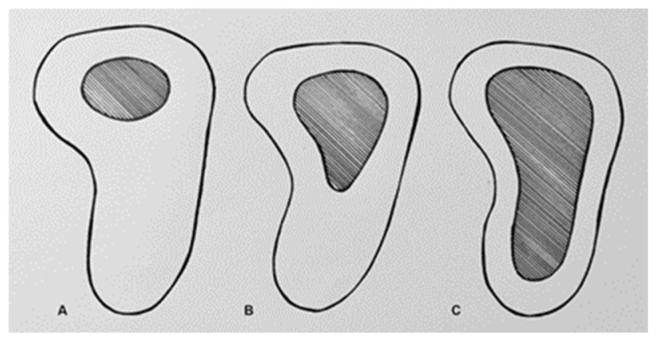
Scaphoid ossification (dark) in relation to the cartilage cap. (**A**) At the age of 6 years. (**B**) At the age of 9 years. (**C**) At the age of 12 years.

In a previously published series, over a hundred pediatric scaphoid fractures were examined in patients with an average age of 12.5 years. The study found that 87% of fractures occurred at the distal pole, 12% at the waist, and 1% at the proximal pole [3]. This was attributed to the ossification pattern (Figure 1).

Between the ages of 9 and 14, all the carpal bones progressively increase in size and undergo ossification [4,5]. By the age of 18, the development of the hand closely resembles that of an adult. Although children are generally at a lower risk of developing scaphoid non-union—due to both the ossification pattern and the typical location of fractures at the distal pole—non-union in younger patients is not uncommon.

Diagnosing scaphoid fractures in children can be challenging, as initial radiographs yield false-negative results in approximately 20% of cases and the clinical presentation is often nonspecific. Only a few clinical signs and physical tests have demonstrated high sensitivity and specificity: anatomical snuffbox tenderness (sensitivity 100%, specificity 98%), supination against resistance (sensitivity 100%, specificity 98%), and longitudinal thumb compression (sensitivity 98%, specificity 98%) [6].

In adult populations, studies have shown a significant difference in the non-union rates depending on the timing of diagnosis and treatment. When treatment is delayed beyond four weeks, non-union occurs in approximately 40% of cases, compared to just 3% when intervention is initiated within the first four weeks [7].

In the pediatric population, scaphoid non-union is primarily due to delayed diagnosis and treatment, although it may also result from surgical failure or compromised vascular supply—particularly in the case of proximal pole fractures [5]. If left untreated, the outcomes of scaphoid non-union in children mirror those seen in adults, including the development of scaphoid non-union advanced collapse (SNAC) and early-onset wrist osteoarthritis [8,9].

Non-vascularized bone grafting—typically from the iliac crest or distal radius—is considered the gold standard for the surgical treatment of scaphoid non-union and is often the first-line approach [10]. However, the failure rates for non-vascularized bone grafting range from 10 to 20%, and the failure rate can reach up to 50% in cases involving necrosis of the proximal pole [11]. Indications for the use of vascularized bone grafting include avascular necrosis of the proximal pole, proximal pole non-union and failure of previous non-vascularized bone graft procedures [12].

Pedicled vascularized bone grafts can be harvested from both the palmar and dorsal aspects of the distal radius. However, the presence of an open epiphyseal plate in pediatric and adolescent patients presents a technical challenge and is considered a limitation of the technique.

This case series presents our experience with a modified and safe volar approach for vascularized bone graft harvesting in the treatment of complex scaphoid non-union in the adolescent population.

## 2. Setting and Population

Our experience is based on three cases of scaphoid non-union in adolescents (Table 1) undergoing surgery from June 2019 to June 2022 at the Unit of Traumatology of Bambino Gesù Children Hospital and Hand and Microsurgery Unit, Jewish Hospital, in Rome, Italy.

All of these three patients were males.

**Figure 2:**
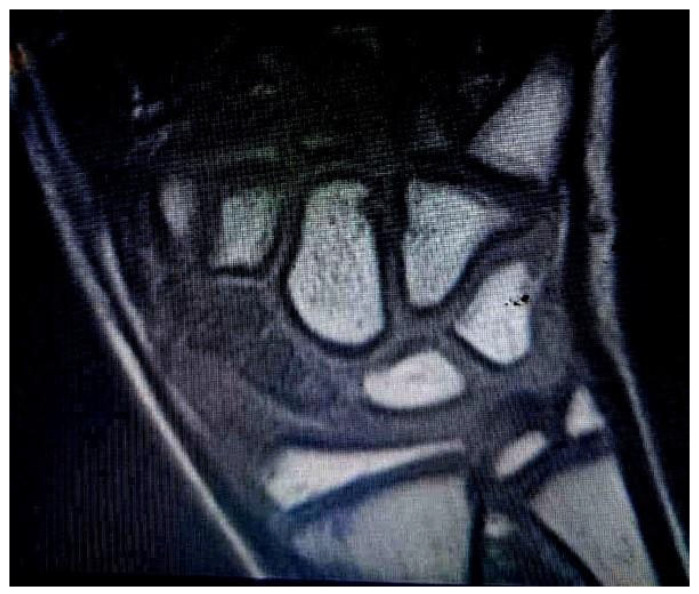
Vascular damage to the whole scaphoid determined via MRI (in the T1 TSE STIR COR sequence) in Patient 1.

Patient 1: A 13-year-old boy presented at the emergency department with wrist pain. X-rays revealed a non-recent fracture of the waist of the scaphoid, which the patient attributed to a trauma that had occurred five months earlier. The patient underwent a preoperative MRI, which showed vascular damage to the entire scaphoid (Figure 2), and a CT scan, which documented bone loss (Figure 4).

**Figure 3:**
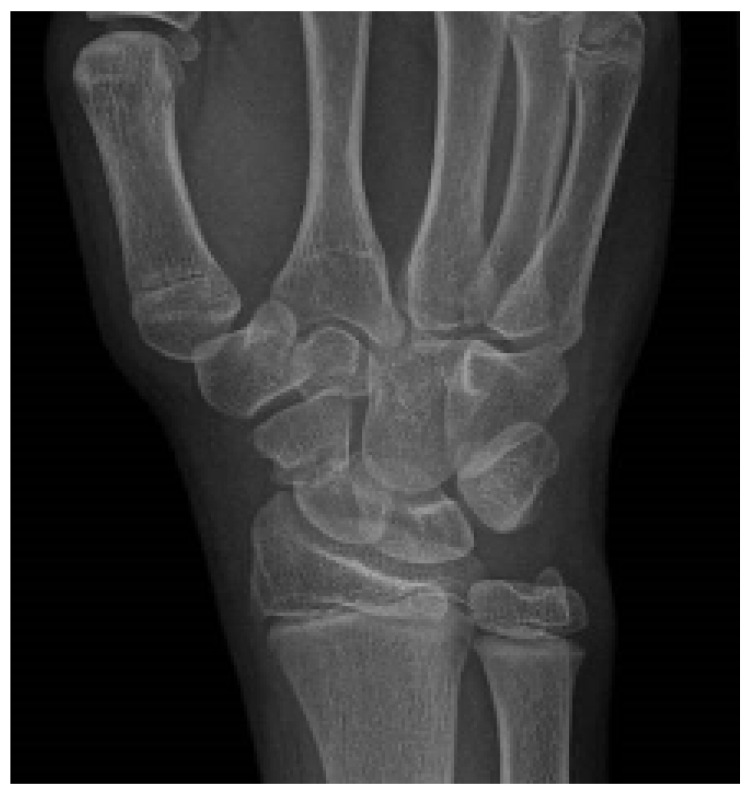
Radiographic evidence of scaphoid waist non-union in Patient 2.

Patient 2: A 13-year-old boy presented at the emergency department with worsening wrist pain, which had started after a fall onto a hyperextended wrist during a five-a-side football game 15 months earlier. At the time of the injury, he did not undergo X-ray, attributing the pain to a minor contusion. X-ray clearly showed scaphoid waist non-union (Figure 3), which was subsequently confirmed by both CT and MRI.

**Figure 4:**
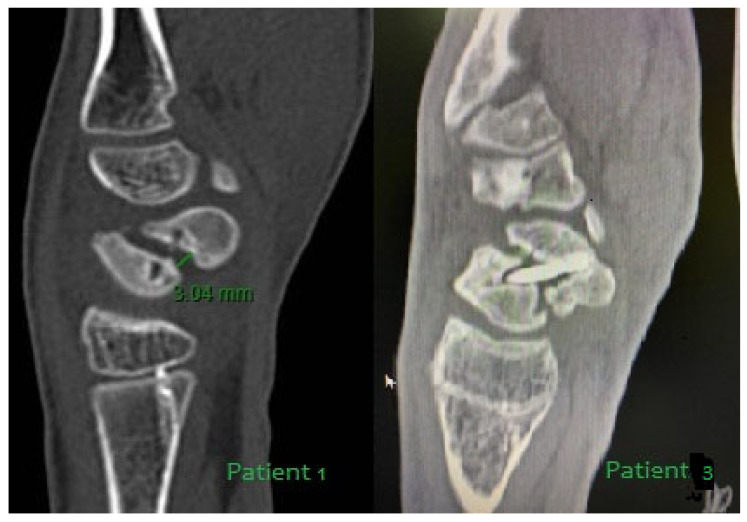
CT scans of Patients 1 and 3 revealed a fracture gap with significant bone resorption. In Patient 3, the non-union had already been fixed with a screw.

Patient 3: A 17-year-old boy sustained a wrist injury during a football match and went to the emergency department, but the X-ray did not clearly show a scaphoid fracture. The patient was not immobilized. Due to persistent pain, after 3 months, he underwent an MRI, which revealed a scaphoid fracture. Conservative treatment with casting was unsuccessful, and six months after the injury, the patient underwent open surgery with debridement of the non-union site and Herbert screw fixation. Although fracture reduction was achieved, the follow-up at eight months showed no callus formation, bone resorption at the fracture site on the CT scans (Figure 4), and ongoing wrist pain. The MRI artifacts on such a small volume of bone tissue as the scaphoid make the vascular assessment difficult. Two years later, the screw was removed and the complex bone defect was treated with a vascularized bone graft.

## 3. Surgical Technique: The Modified Volar Distal Radius Vascularized Graft

The vascularized bone graft harvested from the volar distal radius, commonly referred to as the “Mathoulin technique”, has been well described in the literature [13,14].

**Figure 5:**
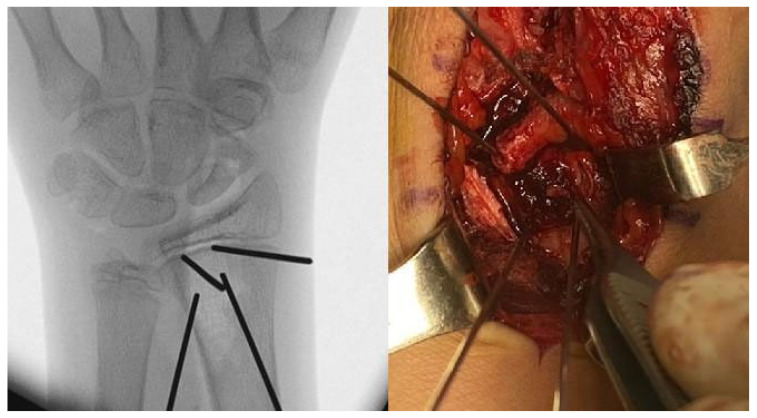
Bone graft demarcation with 4 K-wires to keep the epiphysial radial plate safe.

The vascular pedicle of the graft is the volar carpal artery. The pedicle is identified distal to the pronator quadratus muscle and dissected with the surrounding fascia up to its origin by the radial artery. A key technical aspect of the procedure is that the vascularized bone graft should be harvested proximal to the radial epiphyseal plate. We position four K-wires at the corners of the graft to precisely define its shape, size, and boundaries, given its proximity to the articular surface of the DRUJ and the lunate fossa. Before outlining the graft margins with a small osteotome, we always perform a fluoroscopic check (Figure 5).

**Figure 6:**
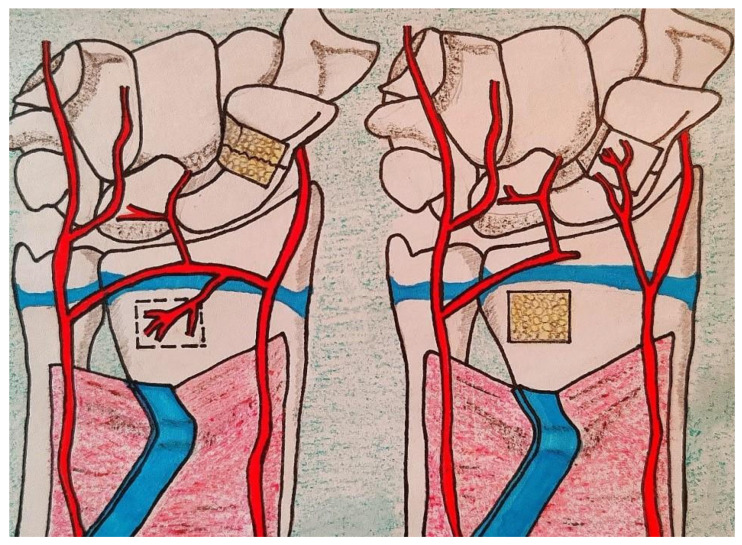
Vascularized bone graft harvesting in children. The bone graft is obtained just beneath the distal radial physis plate.

Once the K-wires are confirmed to be safely positioned away from the epiphyseal plate, a 1 cm^2^ bone graft is harvested using an osteotome or a micro-saw. The graft is then rotated clockwise and inserted into the scaphoid gap (Figure 6). The pseudarthrosis area has already been debrided, and in all the cases in this series, absent or poor bleeding was observed in the proximal pole.

**Figure 7:**
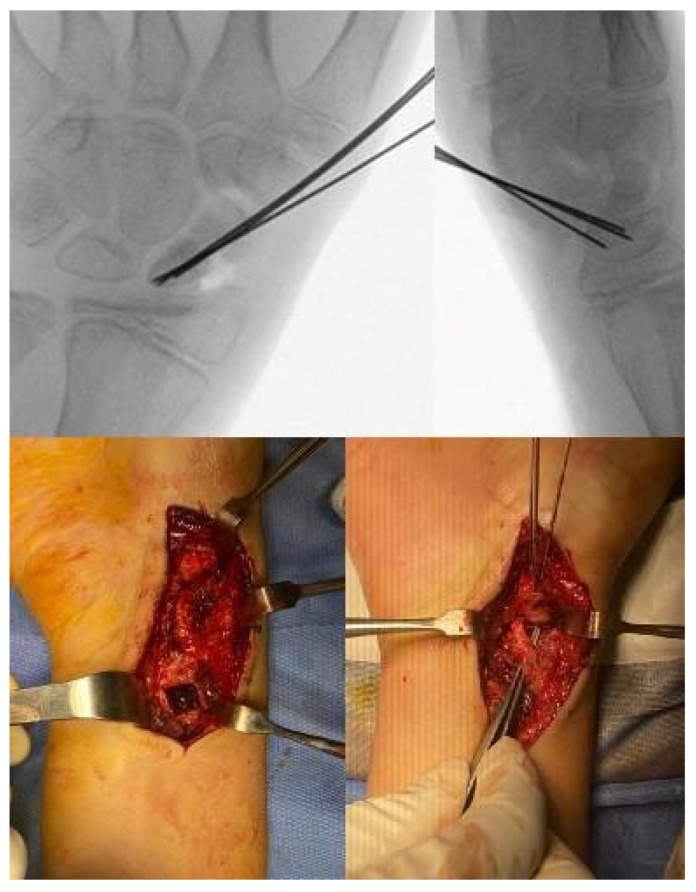
Graft and fracture fixation with K-wires.

The vascularized bone graft is then secured with 2 or 3 0.8 mm K-wires, positioned perpendicularly to the fracture line (Figure 7).

The vascularity is evaluated intraoperatively by observing the graft bleeding after tourniquet release [15,16,17].

The K-wires are buried beneath the skin, and the wrist is immobilized in a short arm cast, including the thumb, for a duration of two months.

Radiographic evaluations were performed at 1, 2, and 3 months postoperatively in this study, with all the scaphoid non-unions demonstrating complete healing at the 3-month follow-up.

Each patient subsequently underwent a minor secondary procedure at approximately three months (3 months ± 8 days) to remove the K-wires.

## 4. Results

Clinical follow-up was conducted through the third postoperative year. The final radiographic evaluation was performed approximately 18 months after surgery. Following cast removal, none of the patients reported radial-sided wrist pain.

**Figure 8:**
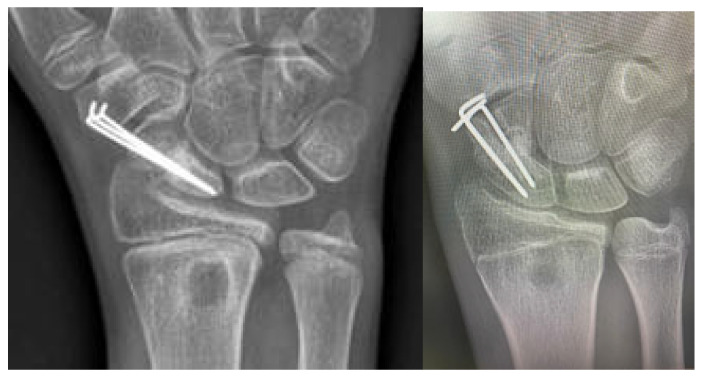
Consolidated fracture at the 3rd post-surgery month of Patients 1 and 3.

Radiographic signs of consolidation at the non-union site were evident by the third postoperative month (Figure 8), at which point each patient began physiotherapy to restore the wrist’s range of motion (ROM)

**Figure 9:**
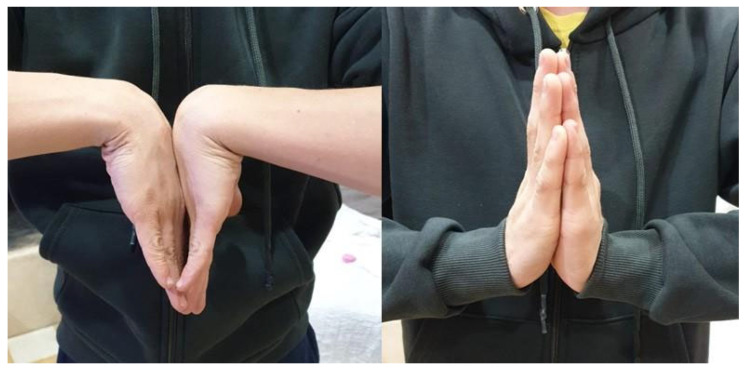
Wrist ROM 12 months after surgery (right hand) in Patient 1.

At the end of treatment, wrist mobility recovery was incomplete, consistent with the severity of the initial injury. One year after surgery, all three patients were pain-free. Two patients presented with a 20° deficit in both wrist extension and flexion (Figure 9), while the third patient showed a 15° extension deficit and a 30° deficit in flexion.

**Figure 10:**
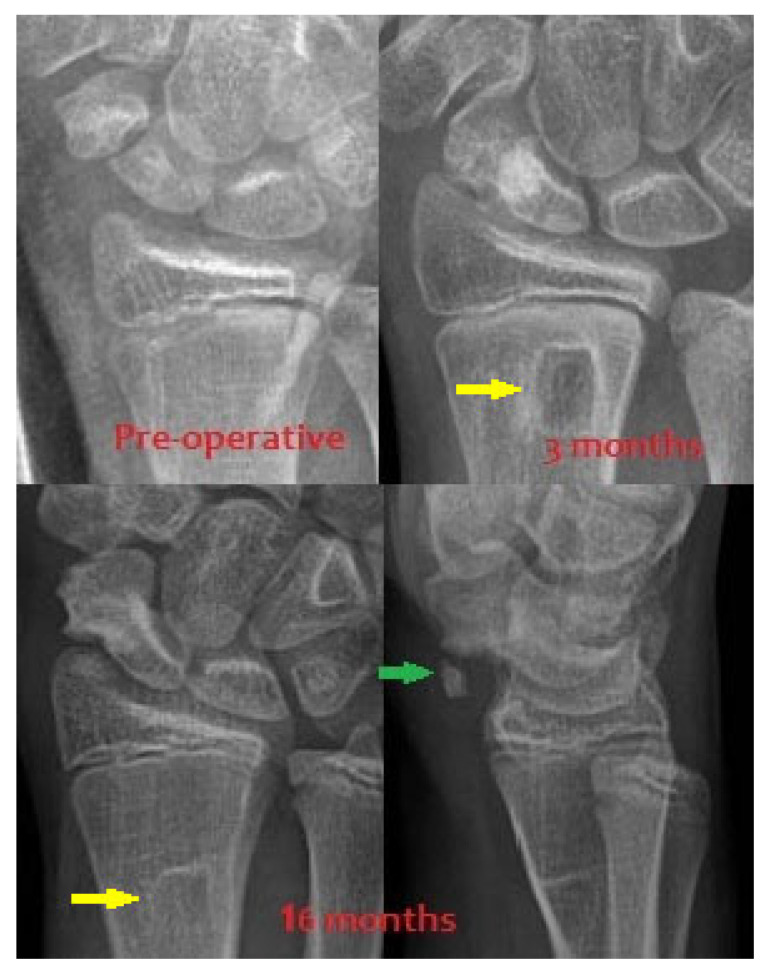
Scaphoid healing process in Patient 1. Top row: Preoperative and 3-month postoperative anteroposterior (AP) projection X-rays. Bottom row: X-rays at 16 months postoperatively. The lateral view shows the ossified pedicle of the vascularized graft (*green arrow*). It causes neither discomfort nor functional impairment and may be surgically removed during the K-wire extraction at the surgeon’s discretion. Also visible is the proximal shift of the graft harvest site due to distal radius growth, indicating that no epiphysiodesis occurred (*yellow arrow*).

All three patients resumed sports activities 12 months after surgery. At the 3-year follow-up, they reported no pain, maintained a wide range of wrist motion (ROM), and experienced no complications (Table 2), including no evidence of radial epiphysiodesis near the graft harvest site (Figure 10). The distal radial defect was completely filled with new bone within one year.

At the 3-year mark, each patient completed the Disabilities of the Arm, Shoulder, and Hand (DASH) questionnaire. All the sections were answered except for the work module, which was omitted due to the patients’ young age, as permitted. The outcomes were favorable, with total disability scores of 5.14%, 13.23%, and 7.08%, respectively.

## 5. Discussion

Scaphoid non-unions are extremely uncommon in children and adolescents. When they do occur, non-unions are most often the consequence of a missed diagnosis or a delayed presentation, frequently because clinical attention is primarily directed toward the distal radius following trauma [16]. The prevention of non-union is the first and most important step in reducing the biomechanical complications associated with an unhealed scaphoid. In accordance with the literature [18], we identified four key points, as summarized in Table 3, that we consider essential for the optimal management of wrist trauma in children and adolescents.

Scaphoid non-union in adults is most commonly treated with non-vascularized bone grafting, which remains the gold standard due to its relative technical simplicity, wide availability, and consistently high union rates, as reported in the literature [19,20]. The rationale for extending this approach to the pediatric population is supported by the robust vascularity of growing bone, which theoretically provides favorable biological conditions for healing. Nevertheless, the literature specifically addressing scaphoid non-union in adolescents remains scarce and limited to small case series [21,22,23,24,25]. Despite the generally favorable biology of young patients, certain scaphoid non-unions in adolescents can present with “critical” features, such as wide bone gaps, compromised vascularity of the proximal pole, or poor bone stock, all of which may compromise the success of conventional non-vascularized grafting. In these complex scenarios, the healing process can be protracted and unpredictable, raising concerns regarding long-term wrist function and the risk of complications such as persistent non-union, carpal collapse, and early degenerative changes. [26]. Our study suggests that vascularized bone grafting can be an effective treatment in selected complex cases with critical bone gaps, such as revision surgery (e.g., the 17-year-old patient) or scaphoid non-union involving a poorly vascularized proximal pole (e.g., the 13-year-old patients). Unlike conventional non-vascularized bone grafts, vascularized grafts remain “alive” and biologically active at the recipient site. The preservation of the blood flow ensures a higher survival rate on the part of transplanted cells, thereby reducing the reliance on creeping substitution for graft incorporation [27]. Creeping substitution is a process of progressive vascular ingrowth, reabsorption and substitution of necrotic bone through which a conventional bone graft is incorporated by the recipient site: a rapid and complete method in cancellous bone, but slow and incomplete in cortical bone. Up to 40–50% of lamellar bone remains necrotic; moreover, mechanical weakening occurs at 6–12 months due to revascularization and reabsorption. The creeping substitution is slower and more incomplete in allografts and heterografts compared to autografts [28]. In vascularized bone grafts, healing is more frequent and rapid in difficult conditions such as avascular necrosis, inadequate vascularity of surrounding tissues, or failed prior graft [29,30]. Based on these considerations, we opted for a vascularized bone grafting technique in our selected patients, modifying the graft harvest site to avoid damaging the growth plate. The use of K-wires in vascularized graft harvesting and fixation is a key point. Delineating the graft with four K-wires is very important for several reasons: it ensures that the growth plate is not damaged, prevents fractures of the DRUJ and radial articular surfaces (as following the best periosteal vessel may position the graft site close to vulnerable structures), and allows for a very precise osteotomy that preserves the periosteum. Fixation of the scaphoid with k-wires is also safer and avoids fragmentation of the delicate graft.

Furthermore, preoperative imaging plays a central role in surgical planning. CT and MRI complement each other, with CT offering superior assessment of the bone morphology and defect size, while MRI provides critical information on the vascularity and early signs of avascular necrosis [16,31].

Table 4 outlines the key criteria used to identify complex scaphoid non-union cases appropriate for this procedure. These criteria, which include prior surgical failure, extensive MRI-documented damage, and proximal pole necrosis, are not mutually exclusive and may often coexist in the same patient. Importantly, while vascularized grafting is technically more demanding and requires careful surgical expertise, it may offer superior biological potential and shorter healing times in selected cases where conventional bone grafting is unlikely to succeed [17].

## 6. Limitations

An expanded study population is necessary to generate more robust prognostic data and to more accurately define the optimal inclusion criteria for this type of graft—such as the patient age range, anatomical location of the non-union, and other relevant clinical variables. Additionally, a longer follow-up period would be valuable to identify potential long-term or currently unknown complications.

Several studies have attempted to compare non-vascularized bone grafts (harvested from the distal radius or iliac crest) with vascularized bone grafts (from the volar or dorsal radius, or free vascularized grafts such as the medial femoral condyle) in order to determine the superior technique. However, none have been able to clearly establish the superiority of one method over another [32,33]. A major limitation across these studies is the lack of a rigorous trial design and randomization. The common methodological weaknesses include small sample sizes, heterogeneity of the fixation techniques (e.g., K-wires vs. screws), unbalanced smoking status among groups, unequal distribution of proximal pole non-unions, and relatively short follow-up periods. Each of these factors introduces bias and limits the comparability of the outcomes. For these reasons, we are currently unable to recommend this treatment over other surgical options with absolute confidence.

## 7. Conclusions

A vascularized surgical approach specifically tailored to adolescents, and free from donor site morbidity, represents a valuable option for the treatment of complex scaphoid non-unions. This technique enables preservation of severely compromised scaphoid bones and may prevent early wrist osteoarthritis in young patients facing delayed diagnosis, substantial bone defects, or previous surgical failure. Although the outcomes of this salvage procedure appear promising, the primary limitation of this study is the small sample size. A larger patient cohort would be required to generate more robust prognostic data. Nonetheless, the observed results—scaphoid healing with rapid recovery, preserved wrist range of motion, and absence of pain during sports activities at the three-year follow-up—are encouraging within the challenging context of pediatric and adolescent scaphoid non-unions.

## Figures and Tables

**Table 1:** Population and setting.

	Age	Months Between Trauma and Non-Union Diagnosis	Edema (% of Bone) and Gap	Previous Surgery
PATIENT 1	13 y	5	100% 2.83 mm	X
PATIENT 2	13 y	18	85% 3.45 mm	X
PATIENT 3	17 y	9	85% 3.92 mm	√ (debridement + Herbert screw fixation)

**Table 2:** Postoperative results.

	Dash Score	Wrist ROM 1 Year After Surgery	Wrist ROM 3 Years After Surgery	Complications
PATIENT 1	5%	20–160°	20–160°	None
PATIENT 2	13%	20–160°	20–160°	None
PATIENT 3	7%	15–150°	15–150°	None

**Table 3:** Recommended approach to prevent scaphoid non-union after wrist trauma in children and adolescents.

Step	Recommendation	Key Notes
Clinical assessment	Thorough physical examination	Snuffbox tenderness and pain with longitudinal thumb compression are highly indicative of scaphoid involvement
Initial imaging	Scaphoid X-ray projection	Improves diagnostic accuracy in suspected fractures
Advanced imaging	CT scan for fracture characterization MRI for occult fracture	Guides the decision between immobilization, percutaneous fixation, or open surgery Detects bone marrow edema in incomplete or nondisplaced fractures
Immobilization	Long arm cast (45–60 days)	Ensures stability and healing; prolonged immobilization is recommended due to limited compliance in pediatric patients

**Table 4:** Main indication.

Primary Indications for Choosing a Vascularized Bone Graft
1. Failure of previous surgery
2. MRI shows damage to more than 80% of the scaphoid
3. Proximal pole necrosis

## Data Availability

The main data concerning this study are available directly in the text. If you need more data, please write directly to the main author.
